# Practitioners’ Experiences of the Influence of Bonsai Art on Health

**DOI:** 10.3390/ijerph18062894

**Published:** 2021-03-12

**Authors:** Caroll Hermann, Stephen D Edwards

**Affiliations:** Department of Psychology, University of Zululand, KwaDlangezwa 3880, South Africa; profsdedwards@googlemail.com

**Keywords:** intervention, ecopsychology, art therapy, mental health, health-seeking behaviour

## Abstract

Bonsai art refers to the cultivation of a miniature tree. This study was motivated by the hypothesis that bonsai art may also be an ecopsychological, therapeutic practice that can have meaningful healing qualities. An international online survey elicited the meaning of bonsai art for 255 skilled bonsai practitioners. Questionnaires and interviews were used to elicit the experiences of participants. The findings supported the hypothesis that, for skilled practitioners, bonsai art was associated with meaningful healing experiences. In particular, the evidence suggests that bonsai art facilitates improved ecological, spiritual and emotional awareness, as well as various healing dimensions, including aesthetic creativity, resilience, adaptability, and social, physical, and personal health. It is viewed as an intervention technique that requires few resources, is easy to apply, and has a minimal impact on any environmental setting. The conclusions drawn point to the ethically sound health promotion value of bonsai art in various settings, such as psychiatric hospitals, retirement homes, rehabilitation centres and prisons.

## 1. Introduction

Recently, practitioners have endeavoured to combine psychotherapy with ecotherapy [1] using non-traditional methods in unconventional and natural settings, such as gardens, parks and wildlife reserves. Ecological psychology refers to the study of the psychological effects of specific biological environments on the psyche of a person [2]. 

Early ecopsychologists believed that people need to reconnect with nature, and ecopsychology has gained popularity because it reconnects people with the wider natural environment, thus enabling them to lead more meaningful and fulfilling lives [3,4]. Ecotherapy uses natural methods and settings to assist with healing, challenging the traditional notion that psychotherapy must be conducted indoors on a couch [5].

To heal is to make a person whole, to transform them from illness to wholeness, from disintegration to integrity. There are various forms of health: physical, personal, interpersonal, social, ecological, and spiritual. There are also different types of energy, which not only correspond to dynamic, human, social, and behavioural patterns, but also respond to correlated environmental resonant frequencies [6]. This study looks at health as a coherent and dynamic integration of all these varying interacting dimensions and contexts [7]. In particular, we focus on the impact that being engaged with nature, more specifically bonsai, might have on bonsai artists’ health.

Integral health depends on the harmony of nature, consciousness, body and soul. The purpose of bonsai is to create a miniature version of a full-size tree in nature. The art of bonsai requires specific skills related to explicit philosophical, horticultural and artistic styles and rules. The skill, effort and creativity of practitioners are rewarded with renewed growth, flowering and fruiting [8]. Very few studies have been conducted on the health advantages of bonsai activities [9,10,11]. The purpose of the present study was to evaluate the influence of bonsai art on health and well-being, as experienced by practitioners themselves. This study elicited answers from established bonsai practitioners regarding the meaning of the practice and the art of bonsai in their personal lives.

### Bonsai as a Form of Ecopsychological Art Therapy

Bonsai means a “tree in a pot” or “tray”. The origins of bonsai are vague, possibly dating back to the Hanging Gardens of Babylon, allegedly built by King Nebuchadnezzar II around 600 B.C. [9,12]. Bonsai art was first practiced in China before it spilled over to Japan. Traditionally, the art form was reserved for the upper classes, where it became steeped in rituals and traditions, such as the tokonoma, which is a dedicated alcove in a home where bonsais are displayed for specific celebrations [8]. Bonsai therefore includes creativity, leisure, and art therapy within one activity, which can have integral health value. 

Ecopsychology studies the unique connection that exists between human beings and the natural world. Robinson [13] refers to ecopsychology as ”soul work”, which strives to awaken, in a creative and appreciative manner, the emotional relationship between individuals and nature, thus resolving mental health issues such as “defeat and loss”. For Fisher [14], ecopsychological foundations include improved contact with nature, the value of positive language and mirroring what is seen and felt in the world. Ecopsychology focuses on awakening an intrinsic appreciation and love of the environment, in which the ecological unconsciousness exists [15]. 

A person experiences numerous benefits from being connected to nature at the time of interaction [16]. It is important to know the client’s affiliation to nature when natural settings are created for patients in therapy sessions [17]. Time spent in and with nature improves psychological wellbeing, ecological awareness and mental health [18]. Additional ethical and ecological concerns need to be addressed when using the outdoors as a form of therapy, especially regarding particularly sensitive environmental terrains and conservation issues. The current state of the planet should be integrated fully into ecopsychotherapeutic practice [2]. 

Contact with the natural environment helps children develop relationships based on cognition, emotional, behavioural, social and biophysical levels. Children’s experiences of nature are important for encouraging imagination and creativity, building cognitive and intellectual development, and social relationships [19]. Art therapy has a long and valid history as a psychological assessment and intervention method. Creative processes are powerful mechanisms in the release of inner emotions [20]. 

Buzzell and Chalquest [5] refer to the healing qualities of nature as a positive experience, facilitating feelings of “being at ease”, “breathing more easily” [5] (p. 89) and being grounded. Nature then becomes the “container” as a layer for “therapeutic holding”. 

Several studies have reported on the restorative health benefits of nature. For example, in one study at a large medium-security [21] UK prison, participants reported that even just viewing natural images helped them to feel calm and normal. The study posited that a restorative environment can restore decreased emotional capabilities. Another study in New York City found that participants who had high exposure to green spaces were associated with a 47% lower likelihood of reporting poor health. The effect of nature on health remains an under-studied subject [22].

Two studies reviewed the physiological effect of “viewing” bonsai trees on patients. Ochiai [23] presented 24 Japanese male spinal cord patients between the ages of 25 and 79 with ten-year-old bonsai trees. The study showed that visual stimulation with bonsai trees was able to elicit comfortable, relaxed and natural feelings, thereby decreasing negative feelings. In another study, Song [24] presented 14 elderly people with bonsai stimuli and found that viewing bonsai induces feelings of relaxation.

## 2. Materials and Methods

### 2.1. Data Collection

Various data collection methods were used. A mixed methods approach, using both qualitative and quantitative techniques, was used. Data were collected through purposeful convenience sampling and global peer recognition of bonsai practitioners. The questionnaires were sent to 300 bonsai practitioners and 255 global bonsai practitioners responded. 

Questionnaire: Well-known and respected bonsai artists (300) were invited to complete an open-ended questionnaire, designed to establish demographics regarding age, gender and bonsai practice. The majority of invitees responded (*n* = 255) to the questionnaire. Bonsai practice was assessed in terms of the level of the participants’ commitment to practising the art of bonsai, as well as the effect bonsai had on their health and well-being.

Interviews: Email correspondence complemented the personal interviews. Semi-structured interviews were followed up with a set of predetermined, open-ended questions that arose from the initial questionnaire. Such semi-structured comprehensive interviews are often employed for qualitative research with groups or individuals [25]. The in-depth interviews included the following research questions, which were posed to the bonsai artist:

*“Please describe in detail the influence that bonsai has on all aspects of your health and wellbeing. Please ensure that you include all aspects of physical, mental, social, spiritual, ecological influences, etc. Please include all your experiences regarding all aspects of health and wellbeing that you can think of”* [9].

The sub-questions consisted of the following:

- “What does bonsai mean to you?”
- “What has bonsai meant in your life?”
- “What influence has it had on your life?”
- Something of a more personal nature: “Has anything in your life ever happened for you to turn to working on your trees to ease the pain, e.g., death of a loved one?”
- “How would your life be without bonsai?” [9].

Instruments

- Spirituality Questionnaire: Participants completed the Spirituality Scale [26], which consisted of 23 items. The scale includes three dimensions, namely self-discovery (a search for meaning), as well as relationships and eco-awareness.
- Generalised Anxiety Disorder Assessment (GAD-7): This instrument is used to identify anxiety [27], with scores of 5 indicating “mild”, 10 indicating “moderate” and over 15 indicating “severe” anxiety
- Physical Health Questionnaire (PHQ-9): This instrument measures depression with subscales of 5 for “mild”, 10 indicating “moderate”; 15 for “moderately severe” and 20 for “severe” depression [28].

The focus of the study is qualitative health, as perceived by the bonsai practitioners themselves. A quantitative objective health assessment of the sample is beyond the scope of the present study. However, to provide some indication of the objective health of the sample, a brief objective report appeared to be warranted.

### 2.2. Ethics

All ethical principles regulating research were observed during the initial doctoral study. Participants were informed of the research protocols, informed consent was obtained during the initial questionnaire and participants were allowed to withdraw at any stage, with their records removed from the study and destroyed. Permission was obtained for the instruments used where necessary. No financial reward was offered to participants. The research proposal was registered with the Research Ethics Committee of the University of Zululand and received approval number C892/14 (UZREG171110-030-RA Dept. 2015/85)

### 2.3. Data Analysis

Data were recorded using the transcription of interviews, and email conversations. Completed questionnaires were transcribed, and saved in an easily coded Microsoft Word document format. Segments of text with related content were sorted into distinct categories. Refined final concepts were categorised into major themes [25]. Themes included information suited to the research question and needs of the project [29]. The thematic analysis [29] involved labelling, analysing, and recording the theme sets in the collected data in rich detail. According to Braun and Clarke [29], themes are not required to be the “most prevalent themes in the data sets but should capture coherent elements of the study”. Eight main themes and nine subthemes were elicited from the participants’ narratives. This process also followed the recommendations of Attride-Stirling [30] and Fereday and Muir-Chochrane [31] in terms of a three-stage approach: (a) an analytical stage comprised of coding material, identifying themes and constructing thematic networks; (b) thematic network exploration, description and summarisation; (c) integration and thematic pattern interpretation. QSR International’s NVIVO 12 was used to explore word frequency and confirmed via text search. Themes were elicited using word frequency and text searches using NVIVO 12. Stemmed words, synonyms, specialisations and generalisations were grouped together. Themes were not ranked in any particular order and content analyses were verified by the second author.

## 3. Results

The simple demographical analysis was based on age and gender, total bonsais owned by participants and, finally, how much time participants spent with their bonsai. Of the 255 participants, 201 were male and 54 were female. This is in line with observations at the club level, where men are traditionally more drawn to the art. Two of the participants were younger than 25, 95 were between 26 years and 40 years of age, the majority (135) were between the ages of 41 and 60 and 23 were older than 61. Bonsai art seems to be attractive to older generations and this trend is also observed at the club level. Three participants owned between one and five bonsais, five owned between six and 20; 67 owned between 21 and 50; 80 owned between 51 and 100 and 100 participants owned more than 100 trees. Forty participants spent between 1 and 2 h per week working on their bonsais, 117 between 3 and 4 h per week, 71 between 5 and 10 h per week and 27 spent more than 11 hours per week on their trees.

Table 1 indicates that the majority of participants fell within the 40- to 61-year-old age range and were male. This is consistent with observations at most club levels. Most of the participants owned more than 50 bonsai trees and spent between 3 and 4 hours per week with their trees.

Table 2 refers to the scores on the Self-Discovery, Relationship and Eco-Awareness sub scales of the Spirituality Scale fall within the upper limits of all subscales and therefore indicate high spirituality. Scores of 5, 10, and 15 are taken as the cut-off points on the GAD-7 for mild, moderate and severe anxiety, respectively. The participants’ scores had a mean of 2.91 and a standard deviation of 3.73, indicating an overall low score for anxiety. The PHQ-9 has similar scores, and 5, 10, 15 and 20 are taken as the cut-off points on the GAD-7 for mild, moderate moderately severe and severe depression, respectively. The participants’ scores had a mean of 2.54 and a standard deviation of 3.33, indicating a low score for depression.

Table 3 illustrates the final major themes and subthemes that emerged from the data analysis. This is followed, in greater depth and detail, by a thematic explication, as reported by particular participants, with sex and age in parentheses. 

Theme 1—ecological awareness: Bonsai appears irrefutably connected to profound awareness and love of all things in nature. Participants reported feelings of empowerment, harmony and respect for nature and conservation. When living close to or in harmony with nature, their behaviour changed. These behavioural changes became an integral part of the artist. The following subthemes were identified:

Respect for nature: A genuine indebtedness to nature is experienced in conjunction with the appreciation of the art of bonsai. Participants felt that, through the art, they were making valuable contributions towards conservation and, in doing so, that they might decrease their negative effect on the Earth.

“With regard to ecological wellbeing, growing Bonsai has made me more aware of the necessity to maintain the growth of trees, jungles and our surroundings.…Growing Bonsai has made me love nature more” (Male, 63).

“Working with Bonsai makes us aware of nature and I think that all bonsai growers are extremely aware of the ecological well-being of nature as a whole. One looks at nature with new eyes and tries to protect our natural heritage” (Female, 41).

Ecological health balance: There is a balance between the emotions that the bonsai artist feels toward the tree and the innate need to create [9]. This need is compared to the idealisation of non-industrialised communities’ ability to live in balance with nature [32]. This contributes to the intangible benefits of happiness and a sense of balance and ecological rootedness. Ecological health balance [33] plays an important role in the interaction between humans and civilisation.

“Having to concentrate when shaping or styling a tree, or keep your mind active when handling a problem tree helps one to stay mentally alert and sharp” (Male, 49).

“A connection to the real world, to changing seasons, to life, to death, and to natural processes” (Female, 55).

“I rate it as my highest priority and place it before my collection. I would rather have the world enjoy nature as it ought to be, regardless of my personal interests” (Male, 62).

Empowerment: Feelings of empowerment relate to the objectives of art therapy, namely “engagement, empowerment and recovery” [32] or “hope, possibility and empowerment” [34], resulting in compassion, empathy and mutual empowerment [34] (p. 224) for participants. Participants unequivocally identified with feelings of control when they cared for the tree, similar to having control over their own lives.

“To make a bonsai trunk taper and the lower branch thicker, while the tendency of a plant is to give most of the nutrient to the top, means that control of the tendency is really necessary in order to achieve a good result (sic)” (Male 32).

“We have to prune the top section of the tree regularly so as to force the nutrient to the lower part. It is an example of controlling the natural tendency in order to achieve a good result. It works both ways, too (in personal life)” (Male, 57).

Theme 2—spiritual awareness: This theme manifested in the subthemes of harmony and order, transcendence and pleasure, contentment, respect, inner spaces and silence and the spirit guide of bonsai. Each subtheme is explicated in its respective order. Spirituality is an integral part of life, and manifests in hopes and connections [35]. Spirituality may also play a significant role in the art of bonsai and is referred to the “soul” of the art of bonsai [36], with some bonsai artists following the more spiritual path of bonsai. Transpersonal approaches to art therapy work on addressing areas of a person’s life or life satisfaction that require improvement, and also recognise “spiritual emergencies” [36] (p. 65) such as a death in one’s family/friendships, stress or illness [37]. The following subthemes emerged:

Harmony and order: Giri [35] claims that humans have the capacity to interact in harmony with the rhythms of the earth. Bonsai artists related this to following horticultural and bonsai styling rules, ancient Eastern traditions and rituals. Although participants recognised the need to follow these rules, even if they were not followed strictly, simply following them to some extent created harmony within the artists. Many participants claimed to follow some sort of tradition or ritual that improved harmony and order for them when styling their trees or walking through their bonsai gardens.

“I have a certain routine… To begin working without the cleaning ritual makes me feel uncomfortable because I could possibly overlook any number of important things” (Male, 64).

“I just concentrated on my tree of choice for a long time. I channeled my imagination toward attempting to design it. When the idea came to me, I started to work” (Female, 36).

“Some of my trees make me think of my mother (Those I don’t like that much) so I carve or change them until I can see myself again” (Female, 56).

Transcendence and pleasure: Participants conveyed feelings of great joy when they were able to spend time in their bonsai gardens with their trees. Subjective emotional states of pleasure were expressed as “happy” and “peaceful moments”. Ballantine [38] labelled the association of “memories… with the scene before one’s eye” as “pleasures of the imagination” [38] (p. 120). Bonsai artists enjoy recreating memories with their trees.

“Bonsai gives me a creative outlet, helps me relax, helps me focus, and sometimes gives a temporary refuge from conflict” (Male, 59).

“A tree also has a soul. A well styled, healthy bonsai gets a higher degree in the next reincarnation. It is already a civilized creature, not a wild one” (Male, 62).

“I make trees of my memories… and people. I remember my Gran, my father and brother who have all passed” (Female, 58).

Respect: The theme of respect, in relation to God and the natural spirit, guides and shapes a bonsai tree. Kandinsky [39] stated that the artist must train his soul as much as his eye. Bonsai creation is borne from the bonsai artist “in a mysterious and secret way” [39] (p. 109) and can mean the difference between a good and a bad creation. This creation is not just a “vague production” (p. 110) of bonsai rules, but has the power to improve and refine the human soul. This is the true “Spirit of Bonsai” (bonsai no kokoro).

“But now I surrender more to God” (Male, 45).

“I let the tree tell me what the best shape is” (Male, 67).

“I like to be quiet and listen for the voice of God” (Male 62).

“I respect nature as it reflects God’s face” (Male, 67).

Contentment: Participants expressed a feeling of contentment when they admired their handiwork. It did not matter if the final product was highly regarded by other viewers or not. The participants did not feel that it was important to be admired by other bonsai practitioners. The private viewing of their own trees brought much happiness, and much pleasure was derived from the object (tree) itself.

“Enjoying the bonsai experience … will help us to better ourselves in many ways” (Male, 76).

“…the artistic significance of bonsai creation makes me delighted to have this opportunity to display a small number of our better examples…” (Female, 28).

“Many feel that being able to spend time with your bonsai, is a divine experience in which every decision to cut back, nip or leave to develop is a reflection of the artist’s spirit and personality” (Male, 59).

Inner spaces and silence: Bonsai practitioners, just like any other artists, also make use of space in between the branches and shadows cast by their tree. It is often said that one should leave a space for birds to fly through the tree. It is believed that some artists may also leave a space for Buddha to sit under their tree [9]. The (bonsai) creation results from the inner experience and insight of the artist [40], and hopefully new attitudes. Milner [40] further states that it is this same inner space that sometimes leads to an inability to (paint) create art.

“During that empty time I got a vision on planting a small banyan tree… It brought joy to my empty time…” (Male 72).

“Negative spaces, which can also be the ‘empty space’ between branches or foliage, are also shaped and proportioned to appear in balance….see completely through the tree’s negative spaces to the background” (Male, 59).

“Negative space resonates within my soul to a certain degree but I focus more on the general ‘feel’ of the tree than on the individual components (as with life)” (Female, 57). “I find this to be key, as I need silence in which to ‘lose myself’ when working on my trees” (Female, 58).

“Negative space is very important for correct balance. As in life, you need the bad to place the good in perspective” (Male, 62).

As an artist works on a tree, be it a personal tree or a tree used during a demonstration, silence is of the utmost importance. Milner [41] found that clients, at times, wish for therapists to be silent. According to Milner [41], silence can be equated to inner emptiness or a gap in which pain can be found. In traditional psychotherapy, silence is often seen as resistance. One study [42] sought to discover the effects of silence on the therapeutic relationship, and found that silence meant that clients had a more pleasant experience. Another study, focusing on experiences of the wilderness, found that silence is an important construct in healthy developing humans [38,43]. Silence is used to explore and gain insight without distraction. Buddhism sees silence not just as the absence of speaking, but as a unification of being. Japanese Noh plays focus on what the actors do not do or how they use space or silence (ma) [44].

“Silence is very important, I even chase my seven-year-old away” (Male, 35).

“Silence allows communion with my trees and results in better concentration” (Male, 65).

“Yes, I valued the silence, especially when I was still working. It was wonderful to return from work and concentrate on my trees with no interruptions or other people who needed attention. Very relaxing” (Male, 42).

Theme Three–Emotional awareness: Nature is considered to be sacred, facilitating the ability to interact with nature’s rhythms, both intellectually and emotionally [35]. Giri [35] also claims that the resonating emotional, spiritual and psychological awareness within humans must be recognised. Being connected to nature promotes emotional well-being.

“Yes, a desire to reduce our pain or to direct our concentration away from a painful thing is normal” (Female, 56).

“Bonsai is like a faithful second wife to me. I can share my love with it. It keeps me busy. I would be sad if anything happened to it. It is really loyal and would never betray me. In the ‘Whole life’ it accompanies me. What is more the first wife will never be jealous of the second one” (Male, 62).

“I have developed coping mechanisms for when a tree dies…When a major tree dies it really hurts me deeply” (Male, 72).

“Part of my personality is inevitably reflected in my trees” (Female, 48).

Theme 4—aesthetic health and creativity: This theme portrays a unique aesthetic and creative health link, specifically art appreciation and creation for its own sake, rather than for fame and fortune. Hagman [45] equates aesthetic experiences with “spiritual oxygen” [45] (p. 10) that is necessary for survival. Aesthetic experiences are unique to each artist and how they engage with the world. These can occur subjectively, knowingly or subconsciously. Aesthetic resonance occurs when the beauty of an object means that it “is worthy of projection” [45] (p. 96), with “worthy” implying that the object resonates with “fantasies (memories) of paradise”.

“It gave me fame, a lot of friends and richness in art appreciation, but I don’t need clubs” (Female, 48).

“It was a big breakthrough, artistically and also financially, to be accepted to exhibit at XXX” (Male 65).

“Growing bonsai engenders a certain degree of love for the tree and pride in the result of our creations” (Female, 32).

Theme 5—social health: Bonsai art is, inevitably, a communal and social activity, ranging from meeting with friends, to small clubs, and beyond. Every person primarily needs to discover their social function within a group or community [41,45]. Bonsai affords the artist a means of belonging and creates a social dialogue [45]. The aesthetic experience (of bonsai) is also tied to the dynamics of social group culture.

“Bonsai brings one together with many people all over the world and so broadens one’s social spectrum. Once they have met, bonsai growers become friends forever” (Male, 58).

“Growing bonsai has made me a member of a large society. I have many friends all over the world. It made me famous as well” (Male 72).

“I think it provides a sense of being part of a community …whom I’ve never met in the flesh but have come to regard as friends” (Female, 48).

Theme 6—physical health: The gardening of bonsai trees is a natural physical form of exercise that can also assist in other physically traumatic situations. Bonsai is a physical and sensory experience, which can improve one’s mental and physical health and well-being [46]. Ecopsychology centres on the link between connectedness to nature and well-being. Hébert [47] claims that our relationship with the environment is developed through our senses. Many researchers have commented on the healing properties of direct contact with nature [13,47,48]

“People who grow bonsai don’t really need to go to the gym on a daily basis. They get enough exercise by moving their trees around, digging up those growing in the ground, going on digs, mixing soil and re-potting their trees regularly. So one can have enough exercise while working with bonsai” (Male, 37).

“It helped me to overcome major (physical) trauma in a positive way” (Male, 42).

Theme 7—resilience and adaptive health: Bonsai implies durability, patience, and a realistic, nature-orientated outlook. Through being conscious of one’s relationship with nature, one becomes aware of an interdependence and connectedness to the world, nature and others. This awareness leads to stability and the capacity to adapt [48].

“…Bonsai has helped me to evolve into a tougher person, more durable in changing situations. I have acquired enough patience to accept the fact that it takes years to create a fine Bonsai” (Male, 48).

“A connection to the real world, to changing seasons, to life, to death, and to natural processes” (Female, 48).

Theme 8—personal health: The art of bonsai offers gentle physical exercises to hard labour when repotting or digging trees from nature.

“Growing Bonsai makes me aware of the need to care about my personal health and wellbeing” (Female, 35).

“Bonsai is giving me a little more confidence in dealing with people. That’s especially significant because I had a life-long speech impediment” (Male, 62).

## 4. Discussion

Practising the art of bonsai allows the practitioner to create, on a reduced scale, what has been seen in nature [8]. The creation of tree and pot form a unified whole. Beauty plays an important role in the transcendent experiences of creation elicited in the practitioner. Ecological awareness is often foremost in most practitioners’ minds, creating harmony, whilst paying homage to nature (and often) conservation. However, “creating” a bonsai means nothing if the survival of the tree is not taken into consideration. The “ecological wellbeing” and “connection to the real world…changing seasons” (male, 63; female, 41; female, 55) of the tree are also important. 

Ecological health balance emerged as the first theme. The need for a balance between public health and ecology is compared to the idealisation of non-industrialised communities’ ability to live in balance with nature [32]. This contributes to the intangible benefits of happiness and a sense of balance and ecological rootedness. Ecological health balance [33] plays an important role in the interaction between humans and civilisation. Feelings of empowerment relate to the objectives of art therapy, namely “engagement, empowerment and recovery” [32] or “hope, possibility and empowerment” [34], resulting in compassion, empathy and mutual empowerment [34] (p. 224) for participants.

The second theme refers to spiritual awareness. Giri [35] claims that humans have the capacity to interact in harmony with the rhythms of the earth. For Chan [8], a tree tells the story “of the fissures in the bark, the bends in the branches… It has not only a past but also a future…”. This connection refers to the “soul” [36] of bonsai. Ballantine [38] labelled the association of “memories… with the scene before one’s eye” as “pleasures of the imagination” [38] (p. 120). Concerning inner spaces and silence, (bonsai) creation results from the inner experience and insight of the artist [38], and hopefully new attitudes. Milner [40] further states that it is this same inner space that sometimes leads to an inability to (paint) create art. As an artist works on a tree, be it a personal tree or a tree used during a demonstration, silence is of the utmost importance. Milner [41] found that clients, at times, wish for therapists to be silent. According to Milner [41], silence can be equated to inner emptiness or a gap in which pain can be found. In traditional psychotherapy, silence is often seen as resistance. One study [42] sought to discover the effects of silence on the therapeutic relationship and found that silence meant that clients had a more pleasant experience. Another study, focusing on experiences of the wilderness, found that silence is an important construct in healthy developing humans [43]. Silence is used to explore and gain insight without distraction. Buddhism sees silence not just as the absence of speaking, but as a unification of being. Japanese Noh plays focus on what the actors do not do or how they use space or silence (ma) [44]. Kandinsky [39] stated that the artist must train his soul as much as his eye. Bonsai creation is borne from the bonsai artist “in a mysterious and secret way” [39] (p. 109) and can mean the difference between a good and a bad creation. This creation is not just a “vague production” [39] (p. 110) of bonsai rules, but has the power to improve and refine the human soul. This is the true “Spirit of Bonsai” (bonsai no kokoro).

For the lay artist, many stories are told through their creations, and they do not need to conform to the rules and “likes” of other people. Following the horticultural and artistic rules of bonsai also brings harmony and order to the practitioner’s life: “when the idea came to me, I started to work” (Female, 36) and “some of my trees remind me of my mother…” (Female, 56), “so I carve and change them until I can see myself again”.

Working with bonsai facilitates emotional awareness and the recognition of feelings. In their theory of transformative coping, Corry, Tracey and Lewis [48] note that art facilitates the discovery of healthy ways to express deep-seated emotions. These aesthetically pleasing experiences feed the practitioners spiritually, as in the abovementioned example of the practitioner who saw her mother in a tree and then made it beautiful for herself. The fourth theme refers to the aesthetic health and creativity of practitioners, where pleasure and enjoyment is experienced to the benefit of the practitioner. Rodin, a nineteenth-century sculptor, stated that “to the artist all in nature is beautiful” [49]. “Yes, a desire to reduce our pain”. 

Concerning the third theme of emotional awareness, Giri [35] claims that the resonating emotional, spiritual and psychological awareness within humans must be recognised. Being connected to nature promotes emotional well-being. Hagman [45] equates aesthetic experiences with “spiritual oxygen” [45] (p. 10) that is necessary for survival. Aesthetic experiences are unique to each artist and how they engage with the world. These can occur subjectively, knowingly or subconsciously. Aesthetic resonance occurs when the beauty of an object means that it “is worthy of projection” [45] (p. 96), with “worthy” implying that the object resonates with “fantasies (memories) of paradise”.

Themes four to eight explicate further differential influences of bonsai art on health. These inclusively extend subjective experiences such as awareness, aesthetic creativity and resilience into relatively more objective social, physical, adaptive and personal health dimensions. Thus, support was provided for the hypothesis that, as an ecopsychological, therapeutic practice, bonsai art provides meaningful healing qualities and promotes integral health. This is an important finding from the point of view of practice, and can be used in various areas of public health. For example, bonsai art has the potential to be of huge benefit as a therapy tool, specifically where the client/patient is separated from the family, primary care group or society. Long-term psychiatric institutions, correctional services, retirement homes, rehabilitation or step-down facilities or orphanages could adapt the intervention for their residents. The therapist should be able to meet with the client/patient in order to assess their therapeutic needs and implement interventions that are suitably graded and tailored to suit participants [9,17].

The results of this research suggested that being involved in bonsai art has both direct and indirect impacts on the well-being and health of individuals. Thus, support was provided for the hypothesis that, as an ecopsychological, therapeutic practice, bonsai art provides meaningful healing qualities and promotes integral health. This is an important finding from the point of view of practice that can be used in various areas of public health. It can be viewed as an intervention technique that requires few resources, is easy to apply, and has a minimal impact on any environmental setting. The conclusions drawn point to the ethically sound health promotion value of bonsai art in various settings, such as psychiatric hospitals, retirement homes, rehabilitation centres and prisons. 

## 5. Limitations of the Study

Certain limitations associated with this study should also be noted, especially those that concern the generalisability and explanation of the findings. For example, the study focused only on experienced practitioners of the art and the results are based on subjective reports by these participants. This study also lacks a control group and pre- and post-test assessments. This, however, paves the way for future studies. On the other hand, the findings do repeatedly indicate, in great depth and detail, that bonsai art had healing qualities for practitioners.

## 6. Conclusions

Bonsai art affords a deep and meaningful insight to be gained into the artist as a creator, the creative act and their continued creation. From a therapeutic perspective, the creation of a bonsai tree affords the artist or client a wonderful experience. The creative process gives meaning and value to what has been created. The practitioner enters into a relationship with the bonsai, creating something new. The constant, cadenced cycles of nature and in time are satisfied through the tending of the tree, providing a form of accomplishment that generates joy in the practitioner. The practitioners’ pain and pleasure with regard to lost or dead trees are overlooked and their exertion when styling and caring for the trees is quickly forgotten, as mothers forget the pain of childbirth. The bonsai becomes a representation that embodies the joy of finding and transcending the self through the creative experience. This study focused on established bonsai practitioners’ experiences of how bonsai made them feel. Bonsai art is an untapped source in the field of ecopsychology and ecotherapy. Further studies will focus on introducing the art of bonsai to non-practitioners in order to make meaningful contributions to emerging forms of psychotherapy. 

## Figures and Tables

**Table 1:** Description of demographics of participants.

		Frequency	%	Mean	SD
Age of participant				2.70	0.638
	>25	2	0.8		
	26–40	95	37.3		
	41–60	135	52.9		
	61<	23	9.0		
Total		255	100%		
Gender of participant				1.21	0.409
	Male	201	78.8		
	Female	54	21.2		
	Other	0	0		
Total		255	100%		
Total bonsais owned by participants				4.05	0.916
	1–5	3	1.2		
	6–20	5	2.0		
	21–50	67	26.3		
	51–100	80	31.4		
	100 and more	100	39.2		
Total		255	100%		
Hours spent per week with bonsais				2.33	0.866
	1–2 h	40	15.7		
	3–4 h	117	45.9		
	5–10 h	71	27.8		
	11 plus hours	27	10.6		
Total		255	100%		

**Table 2:** Includes a summary of the means and Standard Deviation (SD) of the bonsai sample.

		Spirituality Scale				GAD-7	PHQ-9
		Self-Discovery	Relationships	Eco-Awareness	Total Spirituality		
Bonsai sample	Mean	18.71	30.80	59.21	108.72	2.91	2.54
	SD	3.45	2.55	13.81	16.38	3.73	3.33

**Table 3:** Summary of major themes found in narratives of participants.

	Theme	Subtheme
Theme 1	Ecological awareness	Respect for nature
		Ecological health balance
		Empowerment
Theme 2	Spiritual awareness	Harmony and order
		Transcendence and pleasure
		Respect
		Contentment
		Inner spaces and silence
		Spirit guide
Theme 3	Emotional awareness	
Theme 4	Aesthetic health and creativity	
Theme 5	Social health	
Theme 6	Physical health	
Theme 7	Resilience and adaptive health	
Theme 8	Personal health	

## Data Availability

The data are available on the Mendeley server.
